# Unsupervised segmentation of biomedical hyperspectral image data: tackling high dimensionality with convolutional autoencoders

**DOI:** 10.1364/BOE.476233

**Published:** 2022-11-10

**Authors:** Ciaran Bench, Jayakrupakar Nallala, Chun-Chin Wang, Hannah Sheridan, Nicholas Stone

**Affiliations:** School of Physics and Astronomy, University of Exeter, Exeter, Devon, EX4 4PY, United Kingdom

## Abstract

Information about the structure and composition of biopsy specimens can assist in disease monitoring and diagnosis. In principle, this can be acquired from Raman and infrared (IR) hyperspectral images (HSIs) that encode information about how a sample’s constituent molecules are arranged in space. Each tissue section/component is defined by a unique combination of spatial and spectral features, but given the high dimensionality of HSI datasets, extracting and utilising them to segment images is non-trivial. Here, we show how networks based on deep convolutional autoencoders (CAEs) can perform this task in an end-to-end fashion by first detecting and compressing relevant features from patches of the HSI into low-dimensional latent vectors, and then performing a clustering step that groups patches containing similar spatio-spectral features together. We showcase the advantages of using this end-to-end spatio-spectral segmentation approach compared to i) the same spatio-spectral technique *not* trained in an end-to-end manner, and ii) a method that only utilises spectral features (spectral k-means) using simulated HSIs of porcine tissue as test examples. Secondly, we describe the potential advantages/limitations of using three different CAE architectures: a generic 2D CAE, a generic 3D CAE, and a 2D convolutional encoder-decoder architecture inspired by the recently proposed UwU-net that is specialised for extracting features from HSI data. We assess their performance on IR HSIs of real colon samples. We find that all architectures are capable of producing segmentations that show good correspondence with HE stained adjacent tissue slices used as approximate ground truths, indicating the robustness of the CAE-driven spatio-spectral clustering approach for segmenting biomedical HSI data. Additionally, we stress the need for more accurate ground truth information to enable a precise comparison of the advantages offered by each architecture.

## 1. Introduction

Images depicting spatially resolved structural and compositional information about a sample tissue can assist pathologists in disease monitoring and diagnosis [1–4]. A given tissue component or region is generally differentiated from others by i) its constituent molecules and ii) the way in which these are typically distributed in space. This information is encoded in a Raman or infrared (IR) hyperspectral image (HSI) (an image that can be formed by collating Raman or IR spectra acquired at equally spaced out points across a sample), that reveals the presence/quantity of molecular bonds unique to specific molecules at each location [4,5]. These are sometimes also referred to as ‘Raman mappings’ depending on the method of acquisition.

Spectral k-means is a common strategy for segmenting tissue components from Raman/IR HSIs [6–10]. In essence, pixels are grouped together based on the similarity of their constituent spectra. Provided a suitable number of cluster groups are chosen, the resultant cluster map (an image formed by displaying the cluster group assigned to each spectrum in the HSI) reveals where particular molecules reside in the sample, and can be used to segment a tissue into regions defined by their distinct molecular contents. But as stated previously, a given tissue component or region may also be defined by its unique *arrangement* of molecules (i.e. how their constituent spectra are arranged in space). Spectral k-means is inadequate for segmenting tissue regions in this way as it does not use context of the spatial arrangements of neighbouring spectra (separate from the lack of spatial context, the quality of measured spectra may also affect the quality of the outputs [11]). Instead, a more optimal approach would involve i) devising a method for detecting how spectra are arranged in various regions of the HSI and ii) finding a way to cluster regions containing similar arrangements of spectra. Though, the most effective way to detect and manipulate the spatial and spectral features unique to each sample component remains an open question. This is particularly challenging given the high dimensionality of HSI datasets that demand large amounts of memory and long computation times to process.

Recently, deep learning approaches have emerged as promising tools for segmentating HSI data [12]. Compared to ‘classical’ spatio-spectral segmentation approaches such as phasor analysis, extended morphological profiles, and sparse representation models [2,3,12–20], networks learn optimal feature extraction automatically and are therefore more readily applicable to problems where it may be challenging to hand-engineer desirable features and/or consistently extract them from the data. Several architectures have been used for this task, such as recurrent neural networks [21], transformers [22] (both extract long and short range spectral features by treating HSI datacubes as sequences), graph convolutional networks (effective at capturing long range spatial dependencies) [23], and generative adversarial networks [24] (improving network generalisability with an adversarial training scheme). However, architectures based on convolutional networks appear to be the most commonly implemented. All of these have produced promising results on various HSI datsets in supervised or semi-supervised schemes [12,15,25–40]. However, in response to the usual lack of paired training data, effort has been placed towards developing fully unsupervised strategies (including for the separate task of spectral unmixing [41], and virtual staining [42,43]).

Most reported methods for performing segmentation without ground truths utilise conventional analysis approaches as opposed to deep networks [44–53]. However, one approach based on the use of fully convolutional autoencoders (CAEs) stands out in this respect [46,54,55]. Here, the HSI is decomposed into patches and a CAE is used to detect and compress information about spatial and spectral features found in each patch into low-dimensional latent vectors to enable their clustering in an end-to-end fashion (therefore, each stage of the data processing is specifically optimised for the target segmentation task) [54]. This technique belongs to a broader class of approaches that use supervisory signals from a secondary clustering step to enhance the quality of the learned latent representation via a unified learning objective [56–59]. CAEs (as opposed to other architectures such as stacked autoencoders [57,60–64], or more generic autoencoders [65,66]) are well-suited to processing HSI data as they can easily manipulate structured/high dimensional image data, are specialised to extract spatial features, and have a straightforward training procedure. Similar approaches to extracting features with CAEs have been used in [38,40,67–74], but the subsequent segmentation was not learned in a completely unsupervised setting in these cases. Although this approach has been shown to be effective at performing generic image classification tasks [56,75] (and has been applied to satellite HSI data [54], though only achieving relatively poor segmentation quality), there may be room to further optimise the architecture for processing biomedical HSI data. For Raman and IR HSIs, it is the location of peaks/spectral features along a spectrum that encodes information about molecular contents [76], and it is the spatial arrangement of different molecules (represented as unique spatial arrangements of peaks or other spectral features specifically found within a subset of each band in the HSI) that can be used to define a tissue type. Therefore, it is important to ensure that the chosen network is optimised to detect spatio-spectral features that only reside in specific regions of the spectral band. However, generic architectures may not be the most effective at achieving this functionality.

**Fig. 1.**
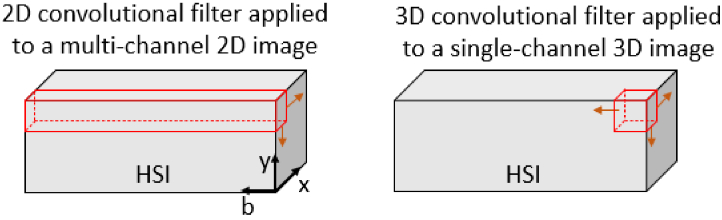
Schematic showing how 2D convolutional filters (red) are applied to multi-channel 2D images, and 3D convolutional filters (also red) are applied to single channel 3D images. 2D filters span the whole spectral band and are ‘scanned‘ across the sample in the x-y dimensions [78,79]. In contrast, 3D filters are typically smaller in the band dimension ‘b’, and take steps in all three dimensions. 2D filters are capable of detecting features that span the whole spectral band (or several features found across the band) but may lack sensitivity to subtle features that may occur only within a specific subset of the band. 3D filters also lack this sensitivity, as they are predisposed to detect more generic features that may occur anywhere within the HSI.

In [54], HSIs are treated as 3D images composed of single channels and processed with 3D convolutional layers [54]. However, 3D convolutional layers are not optimised to detect subtle but important features that may be unique to only a portion of the spectral band, instead they are predisposed to learn filters that detect features that may be present anywhere within the whole input volume. As a consequence, they may miss or be much less efficient at detecting subtle but relevant features that reside in specific portions of the spectral band. Networks composed of 2D convolutional layers are typically used to extract features from multi-channel 2D images [77]. However, these are also sub-optimal, as they are designed to detect features that span the whole spectral band, or simultaneously detect multiple features across the whole band [78,79]. In both cases, the learned representation of patch features may not encode information most relevant to segmentation, spurring a desire for a more efficient feature extraction framework. Figure 1 shows how the filters are applied in both cases, and more details about this problem are provided in Section 1 of Supplement 1.

In contrast to these architectures, the recently proposed UwU-net first processes the input HSI with a series of 2D convolutional layers, ultimately outputting fewer activation maps than the length of the input’s spectral band [15]. The resulting activation maps are then split along the channel dimension, where each is fed into its own ‘inner’ U-Net [80]. Their outputs are then concatenated and processed by another series of convolutional layers to produce the final output. With this architecture, the filters of each inner U-Net are specialised to detect the presence of features encoded in the activation map they are processing. This is unlike generic U-Nets where filters may learn to detect features encoded in all of the activation maps output by the first set of convolutional layers. Experiments have shown that UwU-net architectures outperform more generic U-Nets on supervised image-to-image regression tasks involving HSI data [15]. In these settings, it is clear that specialising feature extraction in this manner provides a clear advantage over generic 2D architectures. I.e. allowing the network to analyse certain activation maps in isolation at some stages (and also allowing it to learn the kinds of features that should be processed by each inner U-Net) appears to improve the network’s ability to detect useful features contained within HSI datasets. Therefore, it is of interest to observe whether this feature extraction framework may confer any advantages for unsupervised segmentation.

### 1.1 Outline of experiments

#### 1.1.1 Synthetic fat/muscle HSIs

First, we demonstrate how unlike spectral k-means, this spatio-spectral approach enables the segmentation of tissue sections containing similar molecular contents by the way their constituent spectra are arranged in space. This is illustrated using synthetic Raman HSIs of porcine tissue divided into sections, each containing a distinct fat distribution. We compare an end-to-end spatio-spectral clustering strategy and the same approach *not* trained in an end-to-end manner with the results of spectral k-means.

#### 1.1.2 Real colon HSIs

To assess our network’s ability to segment more complex samples (requiring the extraction of a much larger set of features), we applied it to real IR HSIs of colon tissue. We show that the resultant segmentations have good correspondence with the contents of HE stained adjacent tissue slices used as approximate ground truths. We also compare our results to those acquired with more generic architectures based on 2D and 3D convolutional layers to assess whether any particular strategy may fail to reproduce major morphological features known to be present in the sample.

## 2. Method: segmenting synthetic Raman HSIs

We applied our UwU-net inspired patch-based clustering approach to synthetic Raman HSIs of muscle tissue divided into sections, each containing a distinct fat distribution. We used two training strategies - an end-to-end training framework, and one that performed feature mining and clustering separately. Here we describe the preparation of the training data and details about the architecture used for training.

### 2.1 Data preparation

**Fig. 2.**
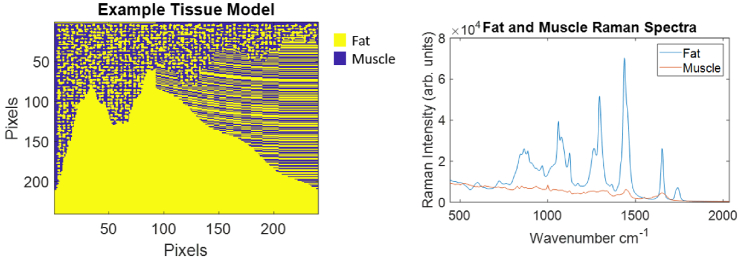
Left: An example 240×240 pixel tissue mask used in the construction of a synthetic HSI. Three different fat patterns are embedded in the muscle - a solid section, a striped section, and a section containing a random distribution of fat ‘globules’. Right: Raman spectrum of porcine fat and muscle samples (back bacon) used to construct synthetic HSIs. These spectra were not pre-processed or normalised, and were acquired with an InVia imaging system (Renishaw, Wotton-under-Edge, UK) with an excitation wavelength of 830 nm, a power of 130 mW, a 50× long working objective, a 600 L/mm grating, and a 3s×10 exposure time.

The synthetic HSIs depict generic porcine muscle tissue with fat distributions designed to mimic simple patterns that may be found in real tissue (though only approximately). The muscle and fat spectra used to construct the HSIs (shown in Fig. 2) were acquired from back bacon samples (with dimensions of approximately 3 cm × 3 cm×3 mm) placed onto stainless steel slides (optical wavelength images of similar samples can be found in [81]). Each of these spectra are single measurements as opposed to averaged spectra. Prior to measurement, samples were stored at 4-6 °C. Measurements were acquired with an InVia system (Renishaw, Wotton-under-Edge, UK) with an excitation wavelength of 830 nm, a power of 130 mW, a 50× long working objective, a 600 L/mm grating, and a 3s × 10 exposure time. The Raman spectrometer was calibrated using a Silicon peak at 520.5 cm^−1^. Two point measurements were taken at two different positions on the same sample, one containing fat and one containing muscle. The spectra were not pre-processed with a baseline correction or smoothing.

Each HSI was constructed from a 240×240 pixel (referred to as the x and y dimensions respectively) binary tissue mask created by assigning fat patterns to different predefined sections (an example is shown in Fig. 2). Each tissue model contained at least one section of solid fat, one section of striped fat, and one section with randomly distributed ‘globules’ of fat. Each fat pixel was assigned the same fat spectrum. Similarly, one muscle spectrum was assigned to each muscle pixel. 800 Raman shift values were used for each spectrum (ranging from 444.2 ${\mathrm{cm}}^{-1}$ to 2035.2 ${\mathrm{cm}}^{-1}$ in steps of 1.98 ${\mathrm{cm}}^{-1}$ ). Therefore, each HSI had dimensions of 240×240×800 . It is evident that the synthetic tissue models only represent highly approximate versions of real tissue. A discussion of i) the effect this has on the robustness of this study and ii) the advantages/disadvantages of using other methods for generating synthetic images are provided in Section 2 of Supplement 1.

Once constructed, each HSI was then decomposed into 3136 20×20×800 patches, by taking steps of 4 pixels in a raster scan style fashion in the x and y dimensions. All negative values were zeroed. Patches were subsequently normalised by dividing them by the max pixel amplitude from the whole HSI. Whole synthetic HSIs are depicted in Fig. S1 in Supplement 1.

### 2.2 Network architecture outline

**Fig. 3.**
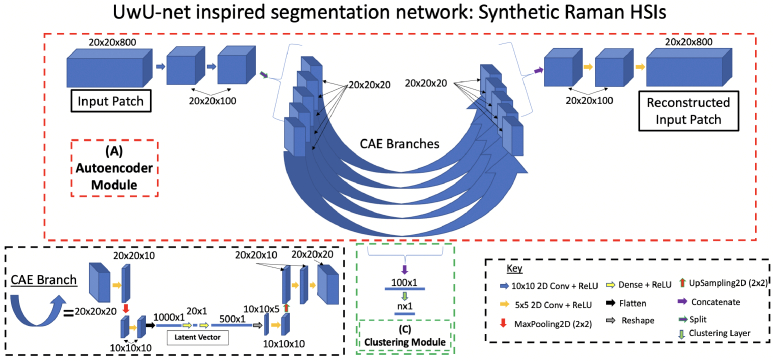
UwU-net inspired segmentation network architecture. The CAE module (A) is used to mine features from the input HSI patch, and compress them into a 100 element latent vector. The architecture is inspired by the UwU-net that is hypothesised to provide a more effective feature extraction framework compared to more generic CAE architectures. The clustering module (C) outputs a probability that the patch belongs to each cluster. With the end-to-end training scheme (A) is initially trained in isolation. After this pretraining step, both (A) and (C) are trained together.

The architecture features two modules, an autoencoder module A and a clustering module C (see Fig. 3).

#### 2.2.1 Autoencoder module

Here, the autoencoder module is used to ‘mine’ features from the input patches that can be used for their subsequent clustering. A CAE learns to i) detect and compress spatio-spectral features contained in each image patch into a set of low-dimensional latent vectors, and ii) subsequently reconstruct the input from this compressed representation. The latent vectors encode information about the spatial and spectral features contained in the input. As a consequence, they can be used to group together patches containing similar contents.

More specifically, the autoencoder module A consists of two components: i) an encoder $A_{E}$ that learns a mapping from an input HSI patch $x_{i}\in X$ to a set of low-dimensional latent vectors $l_{i,m}\in L$ , $A_{E}:X\to L$ were i indexes each patch, and m indexes the latent vector produced by each CAE branch (explained in Section 1 of Supplement 1) and ii) a decoder $A_{D}$ that learns a mapping from the input patch’s set of latent vectors $l_{i,m}$ to a reconstruction of the original input $\hat{x_{i}}\in \hat{X}$ , $A_{D}:L\to \hat{X}$ . Further details about the structure of A and the motivation for its design are discussed in Section 1 of Supplement 1.

#### 2.2.2 CAE+k-means segmentation

Once trained, the set of latent vectors associated with each input image patch will encode information that enables the decoder to perform the reconstruction. This should consist of information about the spatial and spectral features contained in each patch. Regions of the HSI that share similar spatial and spectral features can be segmented by grouping them with patches that have similar latent vectors. This can be performed by i) concatenating the latent vectors produced from each patch into a single 1D vector ( $\bar{l_{i}}$ ), then ii) clustering them with the corresponding $\bar{l_{i}}$ produced from all other patches (e.g. with k-means), and then iii) performing a reconstruction step to acquire the resultant segmentation image. Taking inspiration from [75], we refer to this style of segmentation as **‘CAE+k-means’** clustering (see Section 3.A of Supplement 1). However, with this approach, the CAE does not compress input patches in a way that is optimised for their subsequent clustering, instead, solely prioritising optimal reconstruction quality.

#### 2.2.3 Clustering module and end-to-end clustering

To improve the quality of cluster assignments, the architecture also contains a clustering module C that learns a mapping C:L→O , where $o_{i,j}\in O$ is a set of ‘soft-assignments’, each describing the confidence that the patch (indexed with i ) should be assigned to each of the clusters (indexed with j , where the total number of clusters is a user-defined parameter). The clustering loss is used with the reconstruction loss to update the parameters of A and C to ensure that features are extracted and compressed in a way that optimises the ‘accuracy’ of their subsequent cluster assignment performed by C . In C , the set of latent vectors associated with each input patch are concatenated into a 1D vector $\bar{l_{i}}$ that is then processed by a clustering layer, where the Student’s t-distribution is used to compute $o_{i,j}$ for each $\bar{l_{i}}$ . We call this style of clustering **‘end-to-end clustering’**. Details about how the clustering loss is calculated and utilised in the training process are described in Section 3.B of Supplement 1.

### 2.3 Further details

Further details about the execution of each training scheme are provided in Section 3 of Supplement 1.

## 3. Method: segmenting real IR HSIs

### 3.1 Preparation of real IR HSIs of colon tissue

We applied end-to-end clustering (using the framework described in Section 3.B of Supplement 1) on a set of three colon HSIs from the Minerva dataset [82]. Each image has a corresponding HE slide of an adjacent tissue slice. The three images shown here were chosen based on two criteria: i) they had a large number of glands present (therefore allow us to assess the network’s ability to segment a large set of components), and ii) their corresponding HE slide shows good correspondence with tissue contents as determined with spectral k-means (i.e. their HE slide can be used as an approximate ground truth). In order to enable the most effective assessment of segmentation quality as possible, samples with the highest quality ground truths were selected for presentation in the article.

Colon slices contain several tissue types and components. The most prominent being the intestinal glands, which themselves are composed of several subcomponents: the lumen, epithelial cells, stroma, and nuclei [83]. Given the pixel size of the images ( 5.5×5.5 $\mu m^{2}$ ), it is not clear whether these subcomponents may be resolvable. At the very least, we aim to segment the areas occupied by glands, as well as other neighbouring tissue types that can be clearly observed in the corresponding HE slides.

The HE slide corresponding to each HSI depicts the morphology of an adjacent tissue slice. Therefore, the presence and shape of some components may not exactly reflect those found in the tissue sample depicted by the HSI. With that said, the contents are expected to be similar, e.g. glands should remain clustered in similar regions. Though in some cases, the shapes and number of glands can differ significantly. Furthermore, the HE staining does not reveal all regions that may have different molecular contents, instead highlighting nuclei and the extracellular matrix/cytoplasm to reveal differences in morphology [84]. Ultimately, the HE slides allow us to evaluate whether any architecture fails to reproduce major morphological features of the samples (i.e. whether they generally appear in their expected locations and with similar shapes). Though, it is evident that these slides can not be used to precisely evaluate the quality of the segmentations produced with any of the chosen architectures.

The images were acquired with an Agilent 620 FTIR microscope coupled with an Agilent 670 FTIR spectrometer with a Globar® light source, and a liquid-nitrogen cooled 128 × 128 FPA detector. The resultant images have a $5.5\times 5.5\mu m^{2}$ pixel size with a $704\times 704\mu m^{2}$ field of view. Measurements were acquired in the mid-IR spectral range of 1000–3800 ${\mathrm{cm}}^{-1}$ at a spectral resolution of 4 ${\mathrm{cm}}^{-1}$ . Samples were ‘electronically de-paraffinised’ using a modified extended multiplicative signal correction. The spectral band was truncated to 1000-1800 ${\mathrm{cm}}^{-1}$ , and the spectra were interpolated to ensure each point was separated by 1 ${\mathrm{cm}}^{-1}$ . Further information about sample preparation, image preprocessing, and instrument details can be found in [85].

Images were prepared by taking 10×10 patches in steps of 2 pixels using the same procedure described in Section 2.1. The whole images are depicted in Fig. S2 of Supplement 1.

### 3.2 Learning unsupervised segmentation with three different CAE-based architectures

Three different architectures were applied to the IR HSIs (all sharing the same two-component structure of the architecture shown in Fig. 3, and trained in an end-to-end manner): one where C was a generic 3D CAE (Fig. S3 in Section 5 of Supplement 1), one where C was a generic 2D CAE (Fig. S4), and a UwU-net inspired architecture (Fig. S5). An early stopping strategy was used to determine the stopping point of the CAE pretraining step for the UwU-net and the generic 2D CAE. Each network was trained with Adam as the optimiser, a batch size of 58, and a learning rate of 0.001. Once pretrained, both modules were trained together until less than 0.1% of assignments changed after a given epoch, or after 50 epochs.

The generic 3D CAE architecture was pretrained for 40 epochs, and the maximum number of epochs for the combined training step was set to 20 to limit the total training time to approximately one day. In some cases, the training of the generic 2D and 3D CAEs had to be initiated a few times before the expected exponential decay of the validation loss was observed.

In an attempt to give both styles of 2D networks similar expressive power, the generic 2D CAE network was constructed to utilise a similar number of filters and the same size latent vector as the UwU-net inspired architecture. This was done to ensure any observed differences in their performance could be attributed to differences in the splitting/branching of the layers. The 3D network was coded to have a similar structure as the 2D networks, but featured fewer layers and filters to help reduce computational time. It is not clear how much this may have affected the expressive power of this network relative to the 2D networks, as the architecture is manipulating information about features detected with a 3D convolutional filter as opposed to those detected with a multi-channel 2D filter (i.e. perhaps fewer 3D filters are needed to achieve similar expressive power as the 2D networks). Therefore, it is important to bear in mind that the comparison shown here is not necessarily between two networks with similar expressive power relative to their dimensionality. Nevertheless, the comparison still has some value in the sense that it shows the results that can be produced with either architecture given a clinically relevant time window of 1 day for training (the time between image acquisition and analysis of morphological information should ideally be as short as possible). These network architectures are shown in Section 5 of Supplement 1, while the CAE pre-training loss curves are shown in Section 6.

## 4. Results

### 4.1 Segmentation of synthetic Raman HSIs

**Fig. 4.**
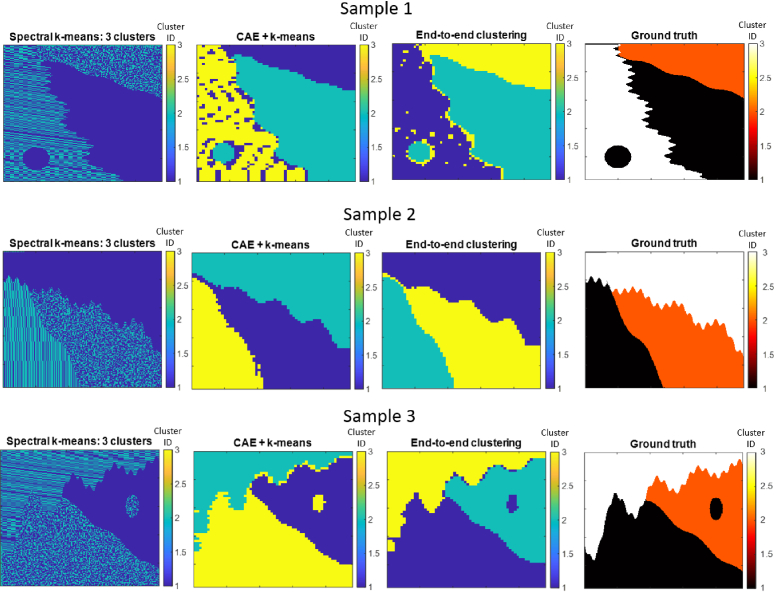
Segmentation results and ground truth for each synthetic HSI. All images shown here have been assigned random colour scales.

As expected (see Fig. 4), spectral k-means was able to accurately differentiate muscle and fat pixels into two distinct clusters (a trivial task, given the properties of each image). The third cluster group is hardly utilised which is expected given i) the algorithm groups pixels by spectral features alone, and ii) there are only two types of spectra in the images (fat and muscle). It is evident that this approach can not be used to segment each tissue section as it does not utilise information about their constituent spatial features. These figures also show the results of our segmentation algorithms (CAE+k-means and end-to-end clustering) that utilise both spatial and spectral features. These were successful in distinguishing each tissue section. A normalised mutual information (NMI) score and adjusted Rand score (ARS) were used to compare the accuracy of the CAE+k-means and end-to-end clustering approaches (as can be seen in Table 1). For Samples 1 and 3, the end-to-end clustering scores were notably higher than the CAE+k-means, and for the remaining sample, the accuracy of both techniques were comparable. The benefits of the end-to-end approach are most clearly observed in Sample 1 in Fig. 4. Here, several regions within the striped section of the tissue are erroneously assigned to the same class as the globule section of the tissue using the CAE+k-means approach. The number of erroneous cluster assignments is significantly reduced in the end-to-end segmentation output. All segmentations are offset from the ground truth by a distance related to the tiling step size that lowers the segmentation quality scores. This is discussed further in Section 4.2.

**Fig. 5.**
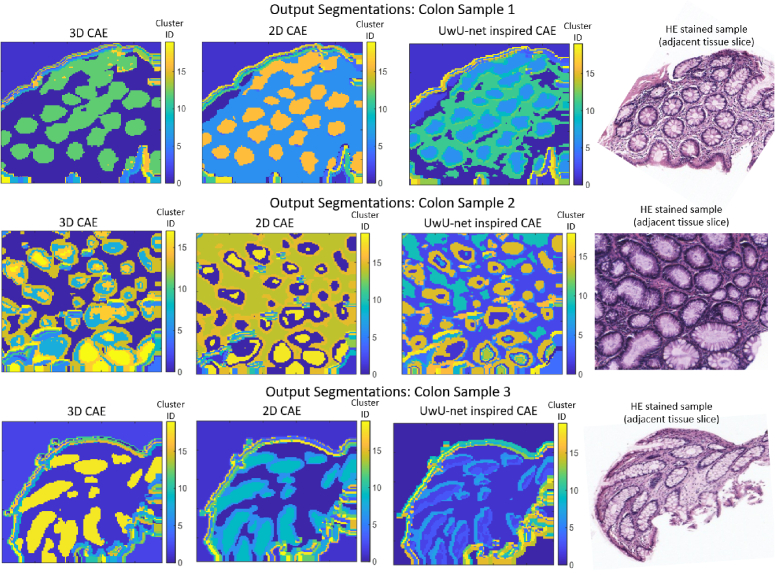
Segmentations of IR HSIs of real human colon samples produced with end-to-end clustering using various architectures. The HE stained adjacent tissue slices used as approximate ground truths are also depicted. All three architectures produced comparable results, and appear to be able to segment the locations of most glands. Though, a precise evaluation of segmentation accuracy is not possible given the approximate nature of the ground truth.

**Table 1.** NMI and ARS scores for the segmentations produced for each synthetic HSI.

Example #	NMI (CAE+k-means)	NMI (End-to-end)	ARS (CAE+k-means)	ARS (End-to-end)
1	0.59	0.68	0.66	0.76
2	0.80	0.81	0.85	0.86
3	0.66	0.71	0.72	0.78

For all samples, patches found near the boundary between two tissue regions were clustered into the same group containing fat globule patches (i.e. their latent vectors were most similar to those representing globule patches). The solid and striped sections have distinct features that are fairly consistent across all patches, whereas there is greater variability and randomness in the features found in globule patches. Therefore it is not entirely surprising that patches with an assortment of different fat patterns adjacent to one another would be grouped into the ‘globule’ class. The extent of this decreased slightly with the end-to-end clustering approach. A smaller patch size might help reduce the spatial extent of these artefacts, though this should be done with caution as each tissue region is defined by spatial patterns that span a certain range, and the spatial context represented in each patch should ideally span this scale.

It is important to note that these results do not indicate how successful this approach may be in more practical settings as our model tissues represent idealised samples for several reasons. These are described in depth in Section 2 of Supplement 1. Furthermore, the few samples shown here do not represent the full range of possible tissue types. Therefore the extent of our approach’s performance on more tissue types remains somewhat unclear. Nonetheless, this experiment demonstrates our UwU-net inspired architecture’s capability to utilise both spatial and spectral information to segment tissue regions primarily differentiated by the way their constituent molecules are arranged in space, and that training it in an end-to-end matter can improve the accuracy of the resultant segmentation for some examples.

There are two other limitations that are important to note. The first is that this algorithm may only be used to segment regions whose defining features span the patch size. Though patches of various dimensions can be easily accommodated by altering the network architecture. Secondly, the accuracy is dependent on the initialisation of cluster centres performed with k-means. If there is significant class imbalance (i.e. one tissue class occupies a smaller area than the others) then this could lead to errors in the resultant segmentation [86].

### 4.2 Segmentation of real IR colon HSIs

All three CAE architectures produced segmentations qualitatively comparable to the corresponding HE stains (Fig. 5). Interestingly, all contained prominent artefacts at the interface between the tissue sample and background - different cluster groups appear to layer on top of each other to form the boundary. We hypothesise that unique latent vectors are required to reconstruct patches containing varying proportions of background and tissue, resulting in the border being defined by a large number of cluster groups and consequently the production of these ‘layer-like’ artefacts. Despite the presence of these artefacts, the border regions appear to maintain the same morphology as that depicted in the HE stain - the boundaries remain smooth, and the shape of the sample remains easy to assess. Though, the various border classes do not appear to correspond with distinct morphological features, and therefore we advise to disregard them when assessing tissue contents.

Given the HE slides depict an adjacent tissue slice and thus only provide approximate information about the morphology of the sample’s components, a precise assessment of segmentation quality (or comparisons between all three architectures) is not possible. Though, it is expected that the morphology of the adjacent slice should share similar structural characteristics as the sample depicted in the HSI (e.g. glands gathered in similar locations). Therefore, the HE stains allow us to assess whether any particular architecture may completely fail to segment any major morphological features expected to be present in the sample. More specifically, a broad measure of segmentation quality may be assessed by whether they contain most of the components found in their corresponding HE slide, and whether these components reside in similar locations.

Each architecture was capable of producing segmentations that satisfy these criteria, demonstrating each has at least some basic ability to segment the larger components present in the samples, and showing the robustness of this CAE-based approach for segmenting real biomedical HSI data. In addition to gland segmentation, the algorithms appeared to differentiate other tissue sections. However, these are not shown in the resultant HE stains, making it challenging to discern whether i) there really are unique tissues present, and ii) whether their boundaries have been accurately segmented. Smaller scale morphological features (e.g. the lumen) may be elucidated by the use of a larger number of clusters as can be seen in Section 7 of Supplement 1. Though without a precise ground truth, it is not possible to evaluate how accurately these objects may have been segmented, or whether they are truly present in each location. Therefore, we focus our attention on large-scale morphological features instead.

The segmentations appear distorted at the bottom and right edges. This is a consequence of how segmentation images are reconstructed (by overlapping tiles from left to right and a top to bottom fashion), and the size of each image patch. A patch placed in the last column will only be overlapped by the patch placed below it, while a patch placed in the final row will only be overlapped by the patch to its right. This produces block-like/smeared artefacts with thicknesses equal to the patch size at the boundaries that can be easily cropped away. Aside from these artefacts, the reconstruction procedure may also subtly translate the position of objects by an amount that appears to be related to the chosen step size. This was observed in the Indian Pines dataset experiment (Section 8 in Supplement 1), and may be easily corrected with additional cropping. This artefact also affected the segmentations produced from the synthetic HSIs mentioned in the previous section, and contributed to lowering the segmentation quality scores.

#### 4.2.1 Using additional cluster groups

It may be possible to elucidate smaller scale morphological features from the colon images by using a larger number of clusters groups. E.g. it appears as though the lumen can be discerned from colon Sample 1 with the use of 40 cluster groups with the UwU-net inspired architecture (see Fig. S10 in Section 7 of Supplement 1). Though without a precise ground truth, it is not possible to assess how accurately these objects may have been segmented. The segmentations produced using additional cluster groups for the other two architecture types are shown as well.

## 5. Conclusion

We have investigated the use of CAEs to perform unsupervised segmentation of Raman/IR HSIs by grouping regions with similar spatial and spectral features. The architectures contain two components: an autoencoder module that mines features from HSI patches and encodes them in low dimensional latent vectors, and a clustering module that then groups similar latent vectors together (akin to grouping together image patches based on the similarity of their constituent features). All of this can be performed/learned in an end-to-end fashion, ensuring the feature mining and compression are optimised for the subsequent clustering step. With a simulation study, we have shown that this approach is particularly useful in cases where different regions of a tissue contain similar molecular contents but may be differentiated by the way their constituent spectra are arranged in space. We also showed how the end-to-end nature of the architecture can improve the quality of the segmentations compared to a strategy that performs the feature mining and clustering in completely separate steps.

To assess whether these approaches can produce segmentations in the face of a much larger feature set that would normally be encountered in real HSI data (as well as experimental artefacts), we tested three autoencoder architectures on real IR HSI data: a generic 2D convolutional autoencoder, a generic 3D convolutional autoencoder, and a 2D architecture inspired by the recently proposed UwU-net. All segmentations were comparable and had good correspondence with HE stains used as approximate ground truths, indicating that this approach can cope with the diverse set of features found in real biomedical HSI data and other confounding experimental factors. Despite the hypothesised advantages of the UwU-net inspired architecture, no significant qualitative differences were observed when compared to the segmentations produced with the generic 2D or 3D CAEs. However, a precise/quantitative comparison between each architecture was not possible given the approximate nature of the ground truth HE stains, which depict an adjacent tissue slice and therefore do not provide information about the exact morphology/boundaries of each relevant component within the HSI data. Therefore, the true extent of the benefits any particular architecture may provide is uncertain. Additionally, the number of samples used in this study is small, and does not represent the full range of different features/sample types that may be encountered in practice (e.g. no tumoral samples were used in this study). Therefore, there remains some degree of uncertainty as to the quality we could expect from using this approach on a wider selection of samples.

It may be possible to use alternative datasets with accurate ground truths to compare architecture performance, such as satellite images of geographical landscapes. However, there are two potential issues with this. The first is that it is not clear whether features residing in specific portions of the spectral bands are as relevant as they are in biomedical HSI data. Therefore, this dataset may not allow us to observe the expected benefit from using any particular architecture for segmenting biomedical HSI data. Secondly, the accuracy of the approach depends on the accuracy of the initialised k-means cluster centres. Poor accuracy has been reported on the Indian Pines dataset and others in [54] and confirmed separately in our own experiment (see Section 8 in Supplement 1). Therefore, it is likely that these kinds of datasets are a poor choice for comparing the performance of our architectures.

Another broader limitation of using this approach is that it is most effective when segmenting regions whose defining features span the patch size. Therefore, it may not be suitable for segmenting tissue regions whose characteristic spatial distribution of molecules spans large distances. Secondly, as mentioned above, the accuracy of the initialised cluster centres strongly determines the quality of the output segmentation. Therefore, this approach may be less effective in cases where there is an imbalance in the representation of tissue sections (i.e. one tissue class occupies a significantly smaller area than the others) [86]. Furthermore, the segmentations suffer from subtle smearing/translation artefacts. Though, these appear to be straightforward to correct by cropping the image using prior knowledge of the patch size and step size.

As it currently stands, the results obtained with each architecture are the first to demonstrate the robustness of the the CAE-driven saptio-spectral clustering approach to segment major tissue components from biomedical HSIs (or arguably any kind of experimentally acquired HSI dataset). Nevertheless, in future work we aim to more precisely quantify the advantages of each architecture by applying them to data with accurate ground truth segmentations, such as more realistic simulated phantoms, or other biomedical HSI datasets. One unexplored feature is the use of the uncertainty encoded in the soft cluster assignments to display information about segmentation quality. This could enable a more rigorous comparison of the effectiveness of different segmentation architectures, or the assessment of how the quality of segmentations produced by different samples may vary from region to region.

## Data Availability

Some of the code used to generate the results shown here can be found in Ref. [87]. Much of this is modified code from [75] (clustering module/end-to-end framework) and [15] (UwU-net inspired architecture). The real human colon IR HSIs acquired as part of the Minerva project [82] are not currently available to the public.

## Supplemental document

See Supplement 1 for supporting content.
