# Supplementary material for: Unsupervised segmentation of biomedical hyperspectral image data: tackling high dimensionality with convolutional autoencoders
Source: Biomed Opt Express. 2022 Nov 10;13(12):6373–88. doi: 10.1364/BOE.476233 (PMC9774878; doi:10.1364/BOE.476233)
Supplement: Supplementary file 1 [file boe-13-12-6373-s001.pdf]

Supplemental Document

Biomedical Optics EXPRESS

Unsupervised segmentation of biomedical hyperspectral image data: tackling high dimensionality with convolutional autoencoders: supplement

CIARAN BENCH,* JAYAKRUPAKAR NALLALA, CHUN-CHIN WANG, HANNAH SHERIDAN, AND NICHOLAS STONE

School of Physics and Astronomy, University of Exeter, Exeter, Devon, EX4 4PY, United Kingdom
*c.bench@exeter.ac.uk

This supplement published with Optica Publishing Group on 10 November 2022 by The Authors under the terms of the Creative Commons Attribution 4.0 License in the format provided by the authors and unedited. Further distribution of this work must maintain attribution to the author(s) and the published article's title, journal citation, and DOI.

Supplement DOI: https://doi.org/10.6084/m9.figshare.21399678

Parent Article DOI: https://doi.org/10.1364/BOE.476233

Supplemental document

Unsupervised segmentation of biomedical hyperspectral image data: tackling high dimensionality with convolutional autoencoders

Here, supplementary information referred to in the main article is presented.

1. DISCUSSION OF ARCHITECTURE CHOICE AND FURTHER NETWORK DETAILS

Here, we discuss the motivation for using the UwU-net inspired architecture and describe the structure of the autoencoder and clustering modules in greater detail.

Information about molecular contents is given by the presence of peaks/spectral features lying within a certain range of the spectral band of the IR or Raman spectra. Thus, it is expected that many of the features relevant to tissue classification will reside in a localised subset of the spectral band. Ideally, the autoencoder module should be optimised to extract these kinds of features to improve efficiency and to increase the chances that all relevant features (even those that may be subtle) can be detected.

Autoencoders processing multi-channel 2D images are usually composed of a series of 2D convolutional layers and pooling layers. However, each filter (dimensions $n \times n \times b$) is predisposed to detect features that may span the whole spectral band, or multiple features throughout the band (decreasing sensitivity to subtle features that reside within a subset of the band). E.g. a filter may learn to detect a subtle feature that occurs at one point in a spectrum alongside a large set of other spectral features. However, the contribution to the activation from this subtle feature could be quite low. Even though a filter may have some sensitivity to it, the resultant activation will not change much if it is present or not if the rest of the features found within the band that the filter detects are also present. With that said, it is possible that the layer may learn filters that only detect features that primarily occupy a specific portion of the spectral band (e.g. having weight values of/near zero everywhere except the region of the band that is of interest), but this is not guaranteed and is likely less efficient than using a more optimised framework for feature extraction. As a consequence, the compressed representation may not robustly encode information about whether subtle spatio-spectral features unique to a subset of the band that are relevant to segmentation are present in a patch.

In other cases, HSI patches have been treated as single channel 3D images and processed by autoencoders composed of 3D convolutional layers [1–3]. However, these are also sub-optimal. The $n \times n \times n$ filters will learn to detect features spanning the filter's volume that may reside anywhere within the input patch, and are not optimised to detect features that may specifically occur in a subset of the spectral band. Though at least here, the resultant activation map will have information about where in the input the detected feature resides in, retaining context about its position along the spectral band (unlike 2D CNNs). With that said, there are no constraints preventing it from mostly detecting generic features that commonly occur anywhere in the volume, potentially missing or being less effective at detecting subtle features that reside within a certain subset of the spectral band. Additionally, 3D convolutional layers often require more processing power and memory to train compared to 2D CNNs, making them generally less appealing to work with.

In contrast to these approaches, the recently proposed UwU-net first processes the input HSI with a series of 2D convolutional layers outputting fewer activation maps than the length of the input's spectral band. The resulting activation maps are then split along the channel dimension, where each is fed into its own 'inner' U-Net [4]. The activation maps produced by the final layer of each inner U-Net are then concatenated together, and processed by another series of convolutional layers to produce the desired output. Therefore, instead of having filters that may

learn to detect hierarchical features composed of features encoded in all of the activation maps produced by the first set of convolutional layers (as would be the case with a generic U-Net), the filters of each inner U-Net are specialised to detect features unique to the activation map used as their input. Empirical evidence has shown that allowing the network to analyse some activation maps in isolation as opposed to all at once (and also allowing it to learn the best way to collate features for further processing by each inner U-Net) provides a clear advantage over generic 2D architectures for image-to-image regression tasks [5]. Therefore, it is of interest to observe whether this feature extraction framework may provide any advantages for unsupervised segmentation.

It is interesting to note that if the first few convolutional layers learn to encode features from various subsets of the input HSI's spectral band into distinct channels (i.e. they learn a straightforward compression along the spectral dimension), then each inner U-Net will learn filters specialised to extract features residing in a specific subset of input HSI's spectral band. This could increase the quality/relevancy of features encoded in the latent representation. Though, given the input patch is initially processed with 2D convolutional layers, there is no guarantee that adjacent channels in the activation maps will encode information about features residing in similar regions of the input patch's spectral band. But, as stated previously, if any of this information is retained, this framework can exploit it. In any case, the architecture could be modified to help preserve this structure in the data, for example, by performing the downsampling along the spectral dimension in a manual pre-processing step with hand-crafted constraints. Though allowing the network to learn the compression in an end-to-end framework (i.e. allowing it to learn which features should be encoded in the inputs to each inner network branch) will ensure that this downsampling is optimised for processing by the rest of the architecture, ultimately improving segmentation quality. Therefore, in this work we consider the fully learned approach.

We adapt this architecture for an unsupervised patch-based clustering approach (see Fig. 3 in the main article). Instead of whole HSIs, the network input is a $(20 \times 20 \times 800)$ patch of the HSI. The input HSI patch is compressed along the spectral dimension to $20 \times 20 \times 100$ as a first step towards reducing the number of parameters required to process/extract spatio-spectral features from the input. These are then split into 5 equal components along the channel dimension (e.g. k_1, k_2, k_3, k_4, and k_5, though any desired number of divisions can be chosen in principle). Each k is then used as the input to its own 'inner' convolutional autoencoder (CAE). The outputs of these parallel inner CAEs are then concatenated together, and processed by a series of 2D convolutional and upsampling layers to produce the desired output. All convolutional layers have a stride of 1.

There are two differences between our architecture and that used in the original UwU-net paper. Firstly, the 'inner' parallel U-Nets have been replaced with CAEs (i.e. essentially U-Nets *without* skip connections). The skip connections are removed to ensure that the network learns how to detect and compress information about patch-defining features into latent vectors. This is not guaranteed with an encoder-decoder style architecture *with* skip connections, as information can propagate through the architecture without passing through the contracting path that forces the network to learn a compressed representation. Secondly, in the original UwU-net paper, the inputs to each inner U-Net is a single activation map. Here, we perform a less aggressive compression, where the input to each inner CAE is a multi-channel 2D activation map. This is done to increase the amount of information available to each inner CAE.

The second module, C (inspired by the clustering module used in [6–8]) takes the latent vectors from each parallel CAE ($l_{i,1}$, $l_{i,2}$, $l_{i,3}$, $l_{i,4}$, $l_{i,5}$, where i denotes the index of the HSI patch being processed) as inputs, first concatenating them into a single vector $\overline{l_i}$, and then processing this with a clustering layer. The resultant output is the probability of the patch belonging to each cluster.

2. ALTERNATIVE METHODS FOR SYNTHETIC IMAGE GENERATION AND COMMENTS ON SYNTHETIC DATA QUALITY

Arranging measured spectra into custom biomimetic patterns is one of several approaches that can be used to simulate Raman HSI data. For example, Raman spectra can be simulated using models of light propagation in digital tissue models [9, 10]. However, it is challenging to ensure these spectra contain realistic experimental artefacts, limiting our ability to assess how our network may cope with real data.

Despite the fact that our synthetic images are composed of real measured data, our chosen construction method has a few disadvantages. It assumes that our sample i) only contains fat and muscle, ii) all fat pixels and all muscle pixels share the same spectra, and iii) each pixel individually contains only fat or only muscle. In real tissue, none of these assumptions may

be completely satisfied. The types of molecules present in a given component may vary from region to region within a sample, producing differences in measured Raman spectra. For example, such changes have been observed in porcine fat spectra acquired at various depths relative to a superficial skin layer, tentatively attributed to the fact that hydrocarbon chains tend to become more ordered (saturated) with depth [11]. Therefore, fat spectra measured from different regions of a pork sample that has been sliced at an angle relative to a skin layer may vary. Physiological parameters like water content (e.g. regions of a tissue slice exposed to air for longer may have less water), and lactic acid [12] vary subtly within a tissue and will have a corresponding effect on measured Raman spectra. Instrument variability may also cause differences in spectra measured from the same kinds of tissue [13]. Furthermore, in an experimental setting it is possible for a probed region to contain both fat and muscle (i.e. at a muscle/fat interface, or thicker samples), whereas each pixel in our simulated HSIs individually contain only fat or muscle [14, 15]. Lastly, bone fragments may be present in some cuts of meat, and so there is no guarantee that substances other than fat and muscle will not be present in a given sample.

However, given this is the first reported demonstration of this approach on any kind of biomedical HSI data, we are primarily interested in showing a proof of concept in an idealised scenario. Our simulated HSIs are sufficient for this task, as i) the data shares the same dimensionality of typical biomedical HSIs (thus offering similar challenges with performing efficient feature extraction), ii) their constituent spectra are of biomedical origin (therefore contain at least some of the features we would expect from real tissue), iii) these spectra contain noise and other experimental artefacts (and therefore allow us to demonstrate the capability of our approach to work in the face of some confounding experimental effects), and iv) their segmentation requires the utilisation of spatial features.

Expanding on point iv), the discrepancy between a purely spectrum-based segmentation approach and one that utilises both spatial and spectral features may not be as drastic in real tissue as may be suggested by the experiments on our synthetic images. Different tissue regions containing the same kinds of molecules may be differentiated by unique spectral features alone due to experimental factors. For example, consider a hypothetical tissue with two regions A, and B. Let us assume that both contain only fat and muscle. Region A has fat and muscle densely packed at a fine scale (e.g. smaller than the area typically illuminated by the Raman probe when used to measure a spectrum), whereas region B mostly consists of muscle with large globules of fat dispersed at a scale much larger than the illuminated region. Because each probed region in A will contain both fat and muscle, the resultant spectra will contain contributions from both. In contrast, most of the spectra acquired from B will be acquired from 'pure' fat or muscle. As a consequence, spectra measured from region A will be distinct from those measured in B - therefore, spectral information alone may be used to segment A. With that said, this represents a hypothetical scenario, and the likelihood of encountering this effect is unclear. Nevertheless, it illustrates how in practice purely spectrum-based approaches may be more effective at differentiating tissue regions than our simulation study suggests. Some of these factors may be effectively replicated with a real physical phantom and should be the subject of future work.

A. Synthetic Raman HSIs

The synthetic images analysed in this work are depicted in Fig. S1. Although the images are composed of 800 channels, only a selection of channels are shown.

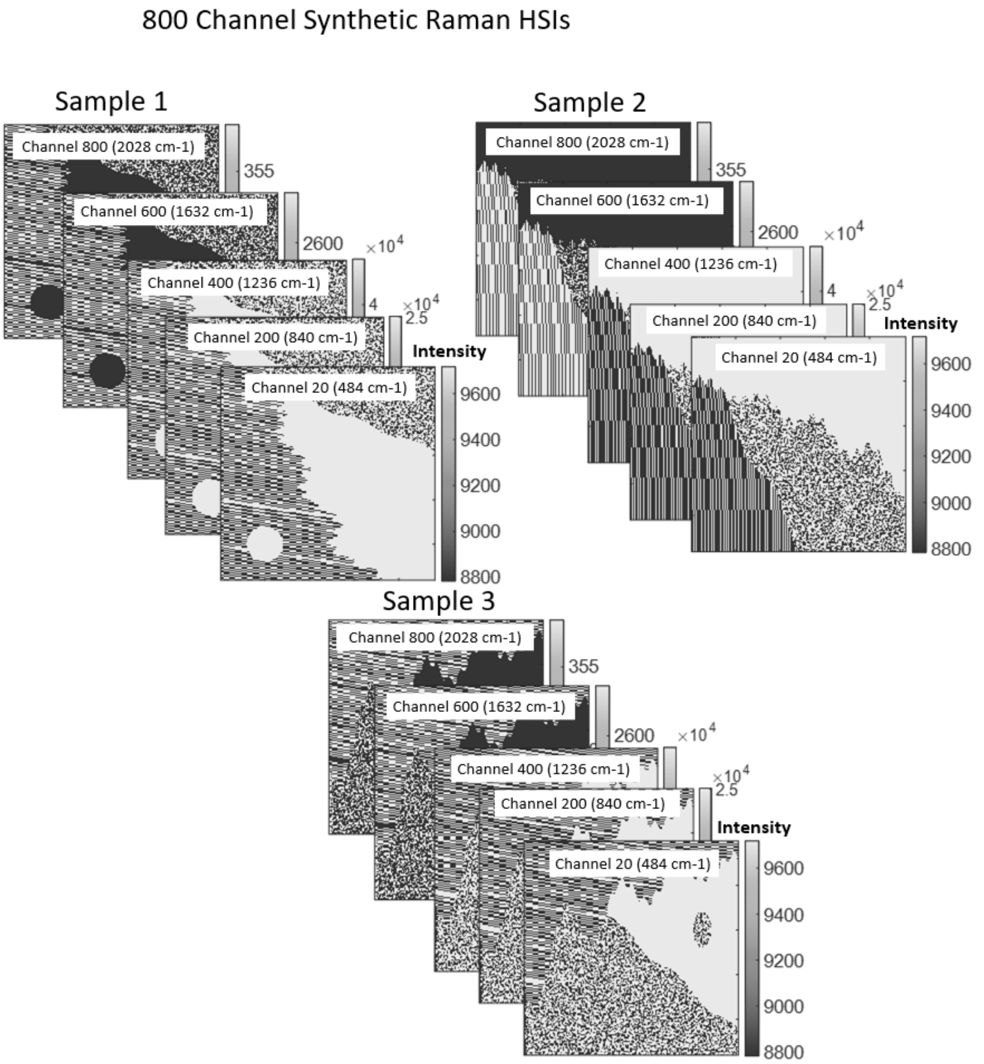

Fig. S1. Synthetic Raman HSIs mentioned in the main article.

3. TRAINING SCHEMES

Here we provide further details about how each training scheme mentioned in the outline in Section 2.2 of the main article is executed.

A. Training details - CAE+k-means

In this approach, the architecture consists only of A that is trained to compress the contents of each patch into latent vectors and reconstruct each patch from these compressed representations. A was trained with the mean square error as the loss function with a batch size of 64, a learning rate of 0.001, and Adam as the optimiser. The HSI of another tissue sample was used as a validation set to monitor the degree of overfitting, ensuring A learned to compress useful features. Training was terminated with an early stopping approach. Once A was trained, the latent vectors for each patch (indexed with i) $l_{i,1}$, $l_{i,2}$, $l_{i,3}$, $l_{i,4}$, $l_{i,5}$ were concatenated into a single 1D vector $\overline{l_i}$. The $\overline{l_i}$ for all patches were then clustered using the k-means++ algorithm in MATLAB R2021b with default parameters, and with 3 clusters. With this approach, the compression and clustering steps are completely separate, therefore, the compression/feature detection learned by A is not optimised for the subsequent clustering step. The pretraining loss curves for most of the networks mentioned in this work are shown in Section 2 in the Supplementary Materials.

B. Training details - End-to-end clustering

With the end-to-end clustering approach the network consists of both A and C. A is initially trained in isolation using the same procedure as Section 3.A. Then, the cluster centres in C are initialised by i) acquiring the latent vector for each patch ($\overline{l_i}$) in the training set with A and then ii) applying the k-means++ algorithm (sklearn) to cluster these latent vectors. Once initialised, all modules are trained together with a weighted loss function (explained shortly). It is suggested in [6] that the unregularised/unconstrained inclusion of the clustering loss may 'distort' the latent representation (i.e. reduce how effectively a patch's spatio-spectral features are encoded in their respective latent vector), negatively impacting the cluster assignment accuracy. Continuing to optimise A during the combined training stage helps ensure that the encoded features can still be used to accurately reconstruct the input, and therefore, robustly encode information about the spatio-spectral features present in each patch. Using autoencoders to strengthen feature representation has also been utilised in [16, 17]. Pretraining A in isolation ensures that the initialised cluster centres are based on robust representations of patch features that have not suffered from this distortion.

Latent vectors are clustered using the Student's t-distribution, which outputs 'soft' cluster assignments [6, 7]:

$$o_{i,j} = \frac{(1 + \|\overline{l_i} - u_j\|^2)^{-1}}{\sum_j (1 + \|\overline{l_i} - u_j\|^2)^{-1}}, \tag{S1}$$

where u_j are the cluster centres (trainable parameters), $\overline{l_i}$ is the latent vector, and $o_{i,j}$ can be interpreted as the probability that the latent vector $\overline{l_i}$ belongs to cluster u_j.

The network is (in part) trained to reduce the discrepancy between the output 'soft' cluster assignments ($o_{i,j} \in O$ where the patch is indexed with i and each cluster with j), and the target soft cluster assignments for each patch ($p_{i,j} \in P$). The target assignments $p_{i,j}$ are computed using,

$$p_{i,j} = \frac{o_{i,j}^2 / f_j}{\sum_j (o_{i,j}^2 / f_j)}, \tag{S2}$$

where j is used to index the cluster centres, and $f_j = \sum_i o_{i,j}$ are the cluster frequencies (number of examples assigned to each cluster). These target assignments strengthen predictions/improve cluster purity, place greater emphasis on cluster labels assigned with high confidence, and prevent large clusters from dominating by incorporating a normalisation [6, 7]. In essence, we leverage an approach similar to self-training where confident assignments are assumed to be more accurate and are used to 'guide' the assignments of patches with similar content.

It is important to note that the use of this clustering module may only improve cluster assignments if the more confident predictions produced with k-means during the initialisation step are accurate. Based on the results acquired with the CAE+k-means approach (e.g. see Fig. 4 in the main article), we believe this assumption to be accurate for our synthetic dataset. The Kullback-Leibler divergence (KL) is used to determine the degree of similarity between the target

assignments P and output assignments O and makes up part of the objective function in the end-to-end clustering approach:

$$L_{Clust} = \mathrm{KL}(P||O) = \sum_i \sum_j p_{i,j} \log \frac{p_{i,j}}{o_{i,j}}. \tag{S3}$$

Two outputs are produced via the forward pass: the cluster assignment for the patch, and a 'copy' of the patch reconstructed from its compressed representation. The model is trained to minimise V, the weighted sum of the resultant reconstruction loss and clustering loss:

$$V = \gamma L_{Clust} + L_{Recon}, \tag{S4}$$

where $\gamma = 0.1$ is a hyperparameter that weights the contribution of the clustering loss to V, and L_{Recon} is the mean squared error.

The target soft-assignments for each patch are updated after each epoch. Training is terminated if one of two conditions were met: either i) once the total number of changed cluster assignments (assessed at the end of each epoch) was less than .1%, or ii) after 10 epochs had elapsed. The latter threshold was chosen to allow for repeat clustering sessions to be completed in only a few hours if necessary. These may be required as the resultant clusters are sensitive to the k-means initialisation, which itself is sensitive to the randomly initialised cluster positions. The network was pretrained with a batch size of 64, a learning rate of 0.001, and with Adam as the optimiser. The CAE pretraining was conducted using the same procedure described in Section A.

C. Training on a single image

In typical deep learning applications, the aim is to train a network so that it generalises well to unseen data (requiring no additional training or modifications to its parameters). Therefore, evaluations of network performance are typically performed by processing unseen test data using a pretrained model. However, we are not interested in finding some pretrained model that is applicable to all images, but rather, a general algorithmic framework that can produce a model that accurately segments any singular image it may receive as an input (akin to how spectral k-means can be applied individually to each image in a given dataset). Therefore, a network's input will consist of patches acquired from a single HSI. Furthermore, the algorithm is run independently and from scratch for each HSI it may receive. The effectiveness of our algorithm is assessed by observing the accuracy of the resultant segmentation produced from this singular HSI.

With that said, overfitting is still something that should be avoided as this will reduce the quality of the compressed representation, and hence, negatively impact the quality of the resultant segmentation. Therefore, we still use a validation loss to assess the degree of overfitting and hence the usefulness of the feature encoding even if we are not aiming to make the network generalisable.

Even though a single image training approach is used here, it would be possible to train a CAE that generalises well to different tissue samples. This could be trained by feeding it a large set of images. After a lengthy training phase, this would ultimately speed up the time needed to acquire segmentations. However, an advantage of our single-image training approach is that the full expressive power of the network is being used to learn the compression and segmentation for the single example. Segmentations produced with a network trained on a large set may be comparable, but will ultimately suffer from some generalisation error.

D. Processing outputs: reconstruction of segmentation images

The procedure for reconstructing each segmentation image is similar to that implemented in [8]. The network is used to assign a particular cluster group (i.e. the cluster group with the highest probability) to each input HSI patch, referred to here as the patch's cluster ID. A 'cluster patch' is produced for each input HSI patch - this is a 2D matrix with the same x and y dimensions as the HSI patch where each element contains the value of the patch's assigned cluster ID. The resulting segmentation map is reconstructed by tiling these cluster patches in an overlapping fashion, placing them in the same location in the x-y dimensions as their corresponding HSI patch. This tiling starts with the HSI patch at the top left corner, moving right along the x-dimension to the end of the row, and then starting again from the left most patch of the next row down and so on until all patches have been exhausted. The corresponding segmentation image will have the same x and y dimensions as the whole HSI.

4. REAL HUMAN COLON IR HSIS

Fig. S2 depicts the real human colon HSIs mentioned in the main article.

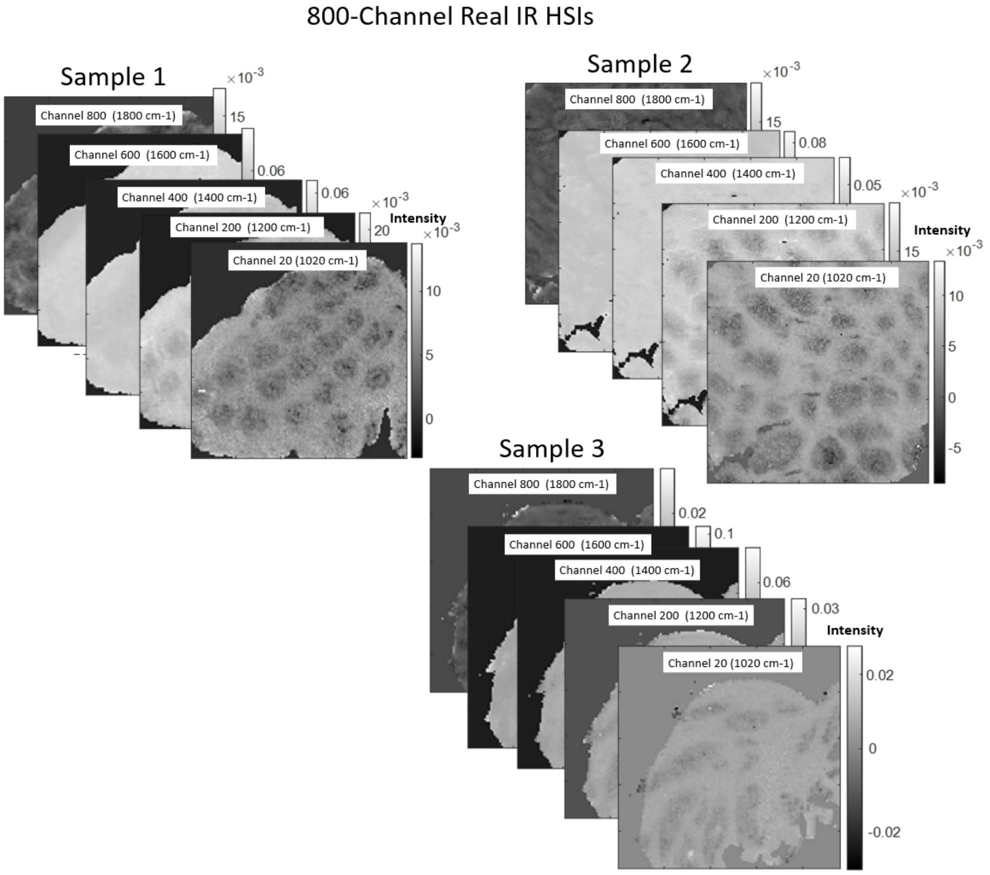

Fig. S2. The real human colon IR HSIs mentioned in the main article.

5. OTHER NETWORK ARCHITECTURES USED IN THIS WORK

In Section 3.2 of the main paper, the segmentations acquired with the UwU-net inspired CAE architecture were compared to more generic CAE architectures based on 2D and 3D convolutional layers. Here we show all three architectures (Figs. S3, S4, and S5). The 2D architecture featured a similar number of convolutional filters and layers as well as the same size latent vector as the UwU-net inspired architecture to ensure that any observed differences in segmentation quality would mostly be a consequence of the use of parallel CAEs. The 3D CAE featured fewer layers and filters to reduce computation time. As discussed in Section 3.2 of the main paper, it is unclear how this may have affected the expressive power of this architecture relative to the 2D architectures given the 3D architecture is utilising information about 3D features.

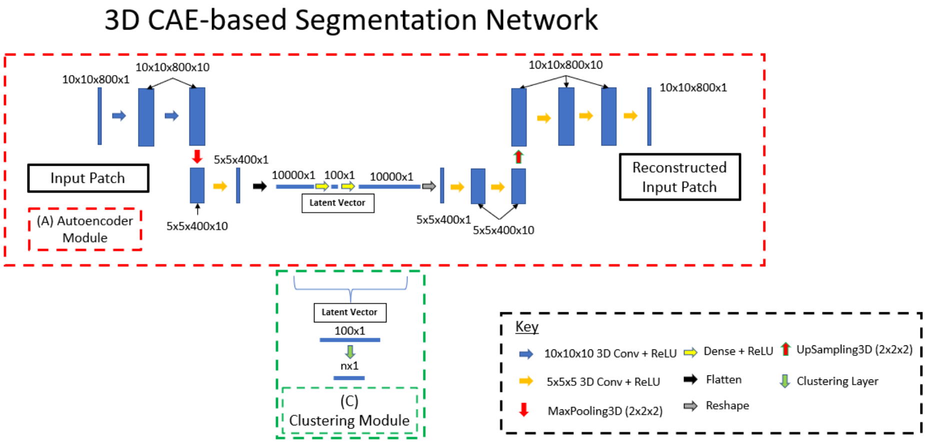

Fig. S3. The generic 3D CNN-based architecture used to segment colon IR HSI images, where C is a generic 3D CAE.

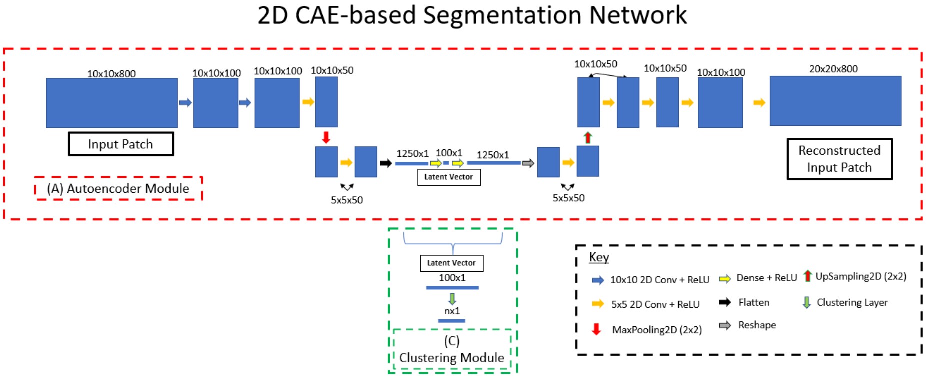

Fig. S4. The generic 2D CNN-based architecture used to segment colon IR HSI images, where C is a generic 2D CAE.

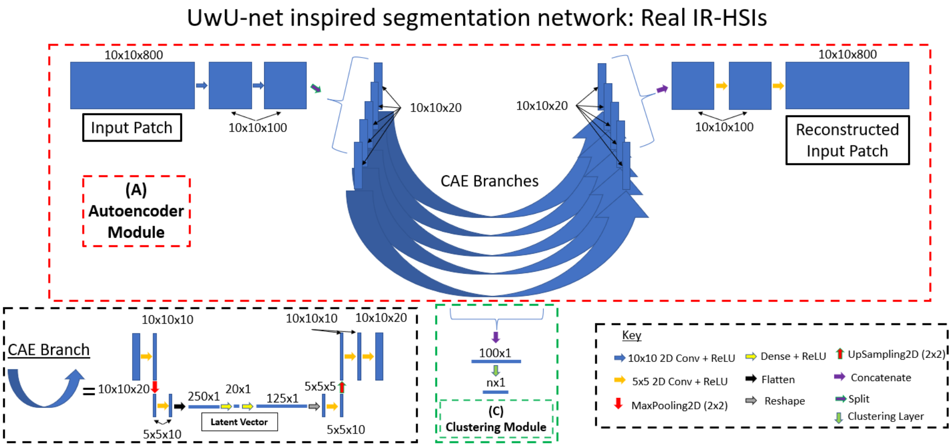

Fig. S5. The UwU-net inspired architecture used to segment colon IR HSI images.

6. CAE PRETRAINING LOSS CURVES

This section contains the loss curves produced at the pretraining stage of most of the networks used in this work.

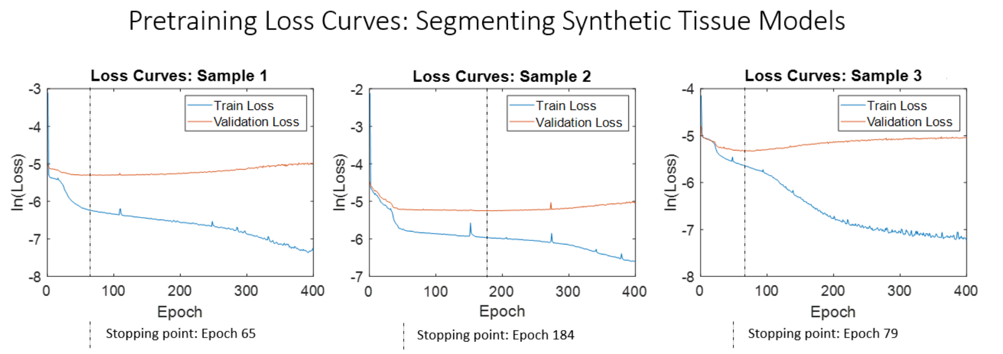

Fig. S6. Validation and training losses for the pretraining of the UwU-net inspired CAE used to segment the synthetic Raman HSIs. The stopping points are shown with dashed lines.

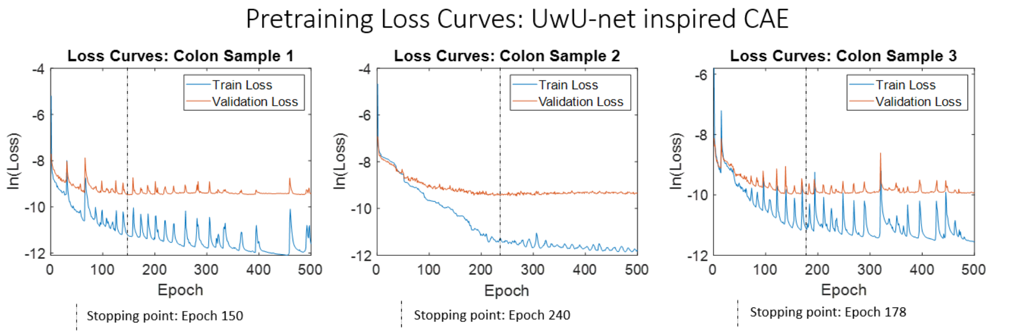

Fig. S7. Validation and training losses for the pretraining of the UwU-net inspired CAE used to segment the colon IR-HSIs. The stopping points are shown with dashed lines.

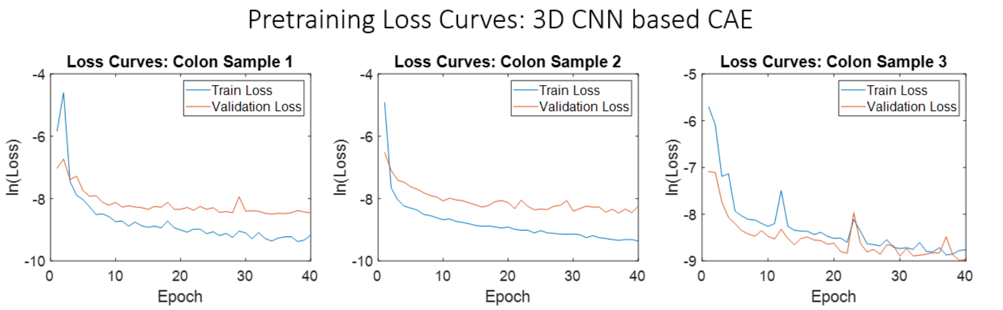

Fig. S8. Validation and training losses for the pretraining of the 3D CAE used to segment the colon IR-HSIs.

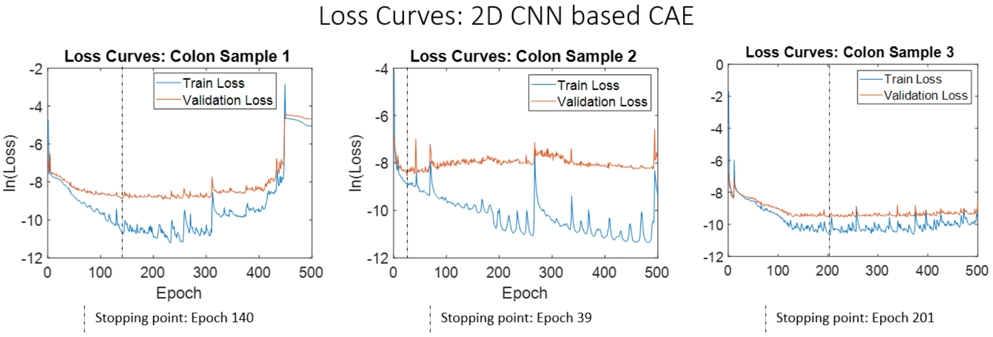

Fig. S9. Validation and training losses for the pretraining of the generic 2D CAE used to segment the colon IR-HSIs. The stopping points are shown with dashed lines.

7. RESULT OF USING ADDITIONAL CLUSTER GROUPS ON A REAL COLON SAMPLE

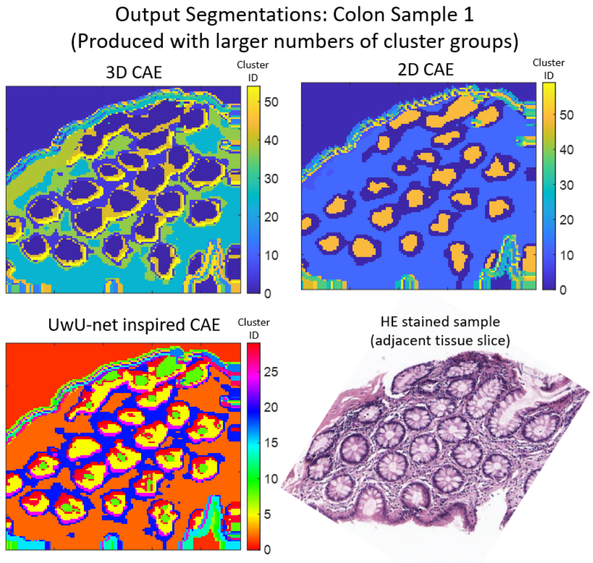

Fig. S10. Segmentations for colon Sample 1, produced with a larger number of cluster groups.

8. SEGMENTION OF THE INDIAN PINES DATASET

The Indian Pines dataset (IP) is often used to assess the performance of HSI segmentation algorithms [8] (https://www.ehu.eus/ccwintco/index.php/Hyperspectral_Remote_Sensing_Scenes). It consists of a geographical landscape scene with 17 classes (including the background) acquired with 200 spectral reflectance bands (where 24 tied to water absorption have been removed) in the wavelength range $0.4 - 2.510^{-6}$ m. At first glance it would seem like a suitable dataset for assessing the performance of our segmentation algorithms. However, this dataset and similar landscape scene HSIs are possibly poor choices for a couple of reasons. The first is that when CAE-based spatio-spectral clustering approaches have been applied to these datasets, the segmentation produced at the initialisation stage has notably poor accuracy (as reported in [8]). This is problematic, as the end-to-end clustering technique works on the principle that the most confident cluster assignments produced at the initialisation stage are accurate. This was confirmed using a UwU-net inspired CAE in our own work, presented below. Secondly, it is not clear whether the satellite HSIs contain the same kinds of features that would be relevant to segmenting biomedical HSIs (e.g. distinct, sharp peaks occurring in specific subsets of the spectral band). Therefore, it may not be suitable to assess the expected performance of each architecture when specifically applied to biomedical HSI data.

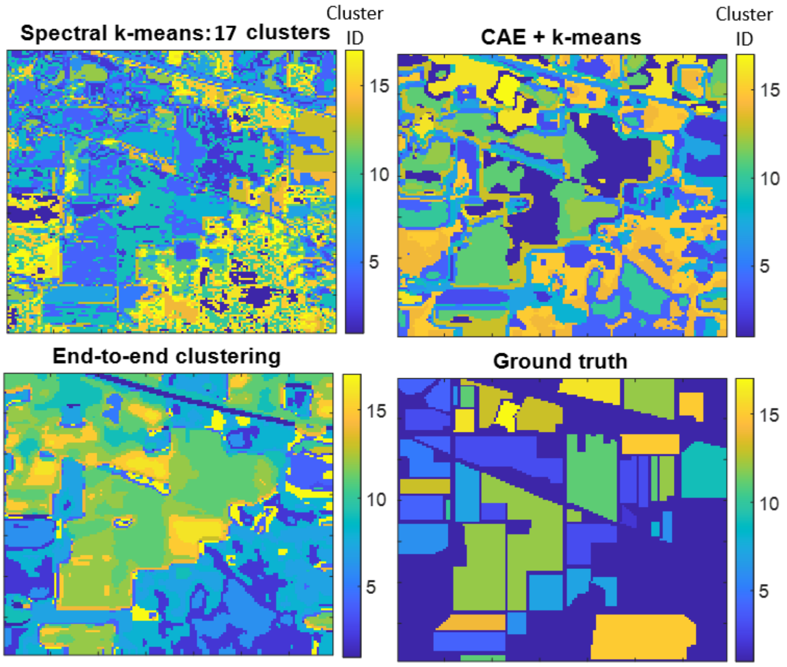

Fig. S11. The results of applying our spatio-spectral clustering approach to the IP dataset (all images assigned randomly indexed colour scales). The resultant segmentation does not effectively differentiate all components of the image. This is verified quantitatively using an NMI score shown in Table S1.

Table S1. Indian Pines Dataset Segmentation Accuracy

NMI Score	CAE+k-means	End-to-end	Spectral k-means
	0.43	0.43	0.42
ARS Score	CAE+k-means	End-to-end	Spectral k-means
	0.23	0.21	0.21

The following describes our own experiment using an UwU-net inspired convolutional autoencoder on the IP dataset. The dataset was cropped to have dimensions $140 \times 140 \times 200$, and then upsampled to give it dimensions of $280 \times 280 \times 200$ (to enable use of 10×10 patches for simplified downssampling without increasing spatial context encoded in each patch). The resulting image was decomposed into 10×10 patches in steps of 2 following the same procedure described in Section 2.1 of the main paper. 12000 patches were used for the training set while the remaining 6496 were used for the validation set. Though, all patches were used in the final evaluation, meaning there was some degree of data leakage in this experiment. The network architecture was the same as that shown in Fig. S5. The autoencoder module was initially trained for 500 epochs with a batch size of 80 (though, not to optimality, as the validation loss was never observed to consistently rise - however, it also showed very little change during the later stages of training, so it is expected that the model is fairly optimised). Then, both the autoencoder and clustering module were trained for 10 updates, or until the number of changed cluster assignments was less than 0.1%. The resultant segmentation was downsized back to 120×120 and cropped to account for the tiling offsetting the segmentation in the x and y dimensions. It is apparent that the resultant segmentation does not accurately differentiate most of the regions within the HSI. The accuracy of the segmentation was evaluated quantitatively using an NMI score, and ARS. Background pixels (whose locations were determined using the ground truth) were not used in the calculation of these metrics. In both cases, spatio-spectral clustering outperformed k-means but both scores are low. The NMI scores were comparable to those reported in [8].

REFERENCES

1. Y. Li, H. Zhang, and Q. Shen, "Spectral–spatial classification of hyperspectral imagery with 3d convolutional neural network," Remote. Sens. **9**, 67 (2017).
2. C. Wang, N. Ma, Y. Ming, Q. Wang, and J. Xia, "Classification of hyperspectral imagery with a 3d convolutional neural network and jm distance," Adv. space research **64**, 886–899 (2019).
3. M. He, B. Li, and H. Chen, "Multi-scale 3d deep convolutional neural network for hyperspectral image classification," in *2017 IEEE International Conference on Image Processing (ICIP),* (IEEE, 2017), pp. 3904–3908.
4. O. Ronneberger, P. Fischer, and T. Brox, "U-net: Convolutional networks for biomedical image segmentation," in *International Conference on Medical image computing and computer-assisted intervention,* (Springer, 2015), pp. 234–241.
5. B. Manifold, S. Men, R. Hu, and D. Fu, "A versatile deep learning architecture for classification and label-free prediction of hyperspectral images," Nat. machine intelligence **3**, 306–315 (2021).
6. X. Guo, X. Liu, E. Zhu, and J. Yin, "Deep clustering with convolutional autoencoders," in *International conference on neural information processing,* (Springer, 2017), pp. 373–382.
7. J. Xie, R. Girshick, and A. Farhadi, "Unsupervised deep embedding for clustering analysis," in *International conference on machine learning,* (PMLR, 2016), pp. 478–487.
8. J. Nalepa, M. Myller, Y. Imai, K.-i. Honda, T. Takeda, and M. Antoniak, "Unsupervised segmentation of hyperspectral images using 3-d convolutional autoencoders," IEEE Geosci. Remote. Sens. Lett. **17**, 1948–1952 (2020).
9. A. P. Dumont, Q. Fang, and C. A. Patil, "A computationally efficient monte-carlo model for biomedical raman spectroscopy," J. Biophotonics **14**, e202000377 (2021).
10. E. Aprà, A. Bhattarai, E. Baxter, G. E. Johnson, N. Govind, and P. Z. El-Khoury, "A simplified approach to simulating raman spectra from ab initio molecular dynamics," arXiv preprint arXiv:1909.03142 (2019).
11. L. B. Lyndgaard, K. M. Sørensen, F. van den Berg, and S. B. Engelsen, "Depth profiling of porcine adipose tissue by raman spectroscopy," J. Raman Spectrosc. **43**, 482–489 (2012).
12. R. Scheier and H. Schmidt, "Measurement of the ph value in pork meat early postmortem by raman spectroscopy," Appl. Phys. B **111**, 289–297 (2013).
13. I. J. Pence, E. Vargis, and A. Mahadevan-Jansen, "Assessing variability of in vivo tissue raman spectra," Appl. Spectrosc. **67**, 789–800 (2013).
14. M. Hedegaard, C. Matthäus, S. Hassing, C. Krafft, M. Diem, and J. Popp, "Spectral unmixing and clustering algorithms for assessment of single cells by raman microscopic imaging," Theor. Chem. Accounts **130**, 1249–1260 (2011).
15. J. Wei and X. Wang, "An overview on linear unmixing of hyperspectral data," Math. Probl. Eng. **2020** (2020).

16. X. Peng, S. Xiao, J. Feng, W.-Y. Yau, and Z. Yi, "Deep subspace clustering with sparsity prior." in *IJCAI*, (2016), pp. 1925–1931.
17. I. Goodfellow, Y. Bengio, and A. Courville, *Deep learning* (MIT press, 2016).
